# Gerstmann-Sträussler-Scheinker syndrome misdiagnosed as cervical spondylotic myelopathy: A case report with 5-year follow-up

**DOI:** 10.1097/MD.0000000000025687

**Published:** 2021-04-23

**Authors:** Liming Cao, Hongye Feng, Xuming Huang, Jiamei Yi, Yanxia Zhou

**Affiliations:** a Department of Neurology, The First Affiliated Hospital of Shenzhen University; b Department of Neurology, The Third Affiliated Hospital of Shenzhen University; c Department of Neurology, Shenzhen Second People's Hospital; d Department of Gastroenterology, Shiyan People's Hospital, Shenzhen, China.

**Keywords:** brain atrophy, cervical spondylosis, Gerstmann-Sträussler-Scheinker syndrome, heterozygous mutation, progressive ataxia

## Abstract

**Rationale:**

Gerstmann-Sträussler-Scheinker syndrome (GSS) is a rare autosomal dominant disease caused by a mutation in the prion protein gene (*PRNP*) that is not well known among neurologists and is therefore easily misdiagnosed.

**Patient concerns:**

: A 49-year-old man was admitted for the first time because of an unsteady walk with mogilalia for 1 year. He underwent a cervical discectomy and a plate-screw fixation 6 months prior, although postoperative gait instability did not improve.

**Diagnosis:**

Whole exome sequencing identified a pathogenic and heterozygous mutation in the *PRNP* 4 years after onset. The patient was eventually diagnosed with GSS.

**Interventions:**

Symptomatic treatment to improve cerebrocirculation and cerebrometabolism was provided.

**Outcomes:**

The neurological decline continued. The Mini-Mental State Examination and modified Rankin Scale scores changed from 19 to 11 and 2 to 5, respectively. Progressive cerebral and cerebellar atrophy on magnetic resonance imaging was observed.

**Lessons:**

Cerebral and cerebellar atrophy are neuroimaging features symptomatic of GSS that become more apparent as the disease progresses. This atrophy is positively correlated with the severity of symptoms and reduced quality of life. Neurologists treating middle-aged patients with progressive ataxia, cognitive impairment or dysarthria, and brain atrophy need to consider the possibility of GSS.

## 1 Introduction

Gerstmann-Sträussler-Scheinker (GSS) syndrome is a rare and fatal autosomal dominant disease caused by a mutation in the prion protein gene (*PRNP*) located on chromosome 20. This syndrome usually manifests as progressive cerebellar ataxia, pyramidal signs, and cognitive decline.^[1]^ It is not easy to distinguish GSS from spinocerebellar ataxia (SCA), spastic paraparesis, frontotemporal dementia, Alzheimer's disease, and Creutzfeldt-Jakob disease (CJD), particularly in the early stages of the disease. Therefore, this condition can be easily misdiagnosed. The prevalence of GSS is 1-10/100 million individuals.^[2]^ GSS was initially discovered through familial transmissions.^[3]^ Dynamic changes in magnetic resonance imaging (MRI) and quality of life in patients with GSS have rarely been reported. The relationship between brain atrophy and the severity of symptoms remains unknown. Herein, we discuss a patient with GSS who was initially misdiagnosed with cervical spondylotic myelopathy (CSM) and followed for 5 years. This case is an example of why GSS should be part of the differential diagnosis.

## 2 Methods

The patient signed the informed consent. The study design was approved by the ethics review board of the 3rd Affiliated Hospital of Shenzhen University (Approval no. 2020-LHYYKY-LW-001).

## 3 Case presentation

**Figure 1:**
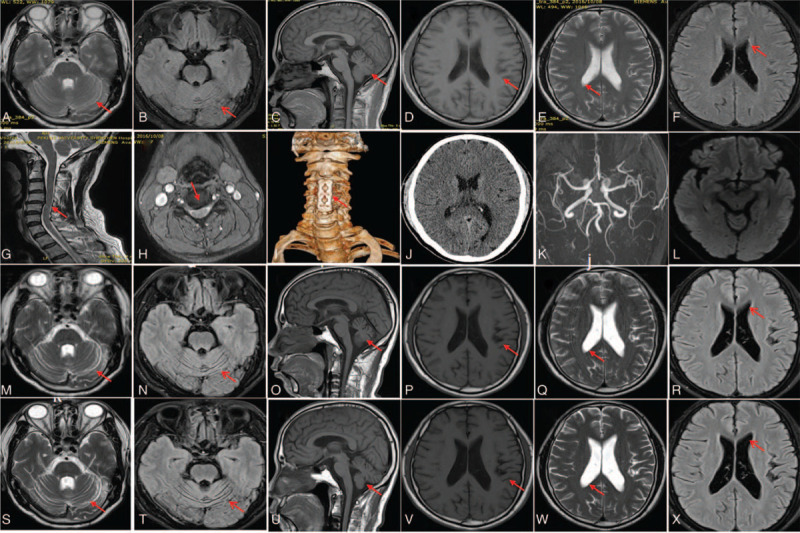
(A–F) Magnetic resonance imaging (MRI) (the sequences are T2-weighted imaging [WI], fluid-attenuated inversion recovery [FLAIR], T1-WI, T1-WI, T2-WI, and FLAIR, respectively) shows no obvious abnormality in October 2016. (G–H) Cervical MRI shows cervical disc herniation at cervical 3/4 (C3/4), C4/5, and C5/6 (arrow) and compression of spinal compression at C4/5 level (arrow). (I) Postoperative cervical vertebra computed tomography three-dimensional imaging shows postoperative changes (arrow) of C3-5 vertebral body and intervertebral space. (J) Brain computed tomography scan shows no obvious abnormality in April 2017. (K–L) Brain magnetic resonance angiography (k) and diffusion WI (l) show no obvious abnormality in February 2019. (M–R) Relatively deep sulcuses in the cerebellar hemisphere (m-o, arrows), insular cortex (p, arrow), and lateral ventricle enlargement (q-r, arrows) on MRI having an aggravating trend in February 2019. (S–X) MRI shows aggravation of cerebral and cerebellar atrophy (arrows) in May 2020 compared with MRI in February 2019.

In April 2017, a 49-year-old man was admitted to our hospital for the first time owing to an unsteady walk with mogilalia for 1 year. In October 2016, CSM was considered for his walking lability. There was no obvious abnormality on the brain MRI (Fig. 1a-f) and there was a cervical disc herniation with spinal cord compression on the cervical MRI (Fig. 1g-h). He underwent a cervical discectomy with cervical interbody fusion cage and plate-screw fixation (Fig. 1i), although his postoperative gait instability did not improve. He had no history of smoking or drinking. His mother was paralyzed at the age of 50 and was bedridden. She died a few years later. His brother had an unsteady gait at about 46 years of age. Physical examination showed a blood pressure of 133/74 mm Hg, clear consciousness, vague speech, impaired cognition (Mini-Mental State Examination score=19/30), decreased muscle strength in both lower extremities (4/5), and cerebellar ataxic gait; he had no other positive signs.

Laboratory analysis in 2017 showed that complete blood count, urinalysis, and routine examination of stools were normal (Table 1). Serum creatinine, myocardial enzyme, electrolyte, C-reactive protein, thyroid-stimulating hormone, fasting blood glucose, glycosylated hemoglobin, and vitamin B12 levels were normal. Fibrinogen level (1.95 g/L) was slightly low and direct bilirubin level (3.8 μmol/L) was slightly elevated. Antinuclear, thyroperoxidase, and thyroglobulin antibodies were negative. A urinary system and abdominal ultrasonography showed no obvious abnormalities. Chest radiograph and brain computed tomography scan (Fig. 1j) showed no obvious abnormalities. Routine tests and biochemistry indicators of cerebrospinal fluid were normal. At that time, he was diagnosed with multiple system atrophy or SCA.

**Table 1:** The second and third hospitalizations of the patient.

	The 2nd hospitalization in February 2018	The 3rd hospitalization in February 2019
Increased or aggressive symptoms and signs	Aggravation of vague speech; diplopia; slowness of pharyngeal reflex; supination orthostatis; and blood pressure changes within normal limits. MRS = 3, MMSE = 15.	Diminution of tendon reflexes in both lower extremities; increased difficulty in walking, requiring assistance; changes in personality and irritation with tension; anxiety; and sleep disorders. MRS = 4, MMSE = 14.
Further examinations	Chest CT showed the left lung bullae, localized pleural thickening, multiple mediastinal lymphadenopathy. Sensory nerve action potential was not evoked in sural nerve, and the latency of motor latency at bilateral tibial nerve was prolonged. Electroencephalogram was normal. Repeats of CAG in spinocerebellar ataxias 1, 2, 3, 6, 7, 12, DRPLA genes were in normal range.	Carotid ultrasound showed formations of the bilateral carotid plaque. MRI showed no obvious abnormalities on brain magnetic resonance angiography (Fig. 1 k) and DWI (Fig. 1l), worsened brain atrophy (Fig. 1 m-r). All exons sequenced by high-throughput sequencing identified a pathogenic and heterozygous mutation in prion protein gene: Exon 2, c.305C>T, p.(Pro102 Leu) (Fig. 2)
Treatment and outcome	Intravenous vinpocetine 30 mg/d and oxiracetam 4 g/d; oral butylphthalide 0.6 g/d, vitamin B12 1.5 g/d and B1 30 mg/d. The symptoms showed no obvious improvement.	The same as the 2nd hospitalization

The treatment plan included improving cerebrocirculation (intravenous vinpocetine 20 mg/d and oral butylphthalide 0.6 g/d) and cerebrometabolism (oxiracetam 4 g/d); symptomatic treatment was provided. His symptoms showed no obvious improvement, and he was discharged on day 9. The modified Rankin Scale score was 2.

The details of the second and third hospitalization of the patient are illustrated in Table 1 (Fig. 1k-r).

The *SNCA* gene exons were detected by multiplex ligation-dependent probe amplification (Kingmed Center for Clinical Laboratory, Guangzhou, China), and no gene mutations were noted. We found that he had a heterozygous mutation in exon 2 of the *PRNP* gene, positioned on chromosome 20. Specifically, he had a missense mutation of substitution of cytosine with thymine at gene loci 305, which caused proline-to-leucine substitution (P102L) on the protein polypeptide chain. The patient was eventually diagnosed with GSS in February 2019. The patient was hospitalized for the fourth time after fifteen months. The main symptoms were dysdipsia, dysarthria, dizziness, dysphasia, and diplopia, and chief signs were horizontal nystagmus, shallow nasolabial sulcus on the right side, tongue muscle fibrillation, decrease of muscle strength on upper and lower extremities, decreasing muscle tone, absence of tendon reflex. MRS and MMSE scores were 5 and 11, respectively. Repeated MRI revealed worsening of the cerebral and cerebellar atrophy (Fig. 1 s-x); diffusion-weighted image (DWI) reexamination showed no abnormalities. Based on the third hospitalization, oral alprazolam 0.4 mg/d and duloxetine 120 mg/d were added to the treatment regime. Sleep disorders improved, but other symptoms showed no improvement. The patient was satisfied with the diagnosis and treatment.

## 4 Discussion

The average age at onset of GSS is 49.5 ± 4.51 years (range: 48–52 years).^[4]^ The duration of the disease ranges from 1 to 10 years.^[5]^ GSS is usually an autosomal dominant inherited disease, thus, a single allele mutation can increase the risk of GSS. Patients with heterozygous mutations have a 50% chance of passing abnormalities in their DNA sequence on to their descendants in every birth.

**Figure 2:**
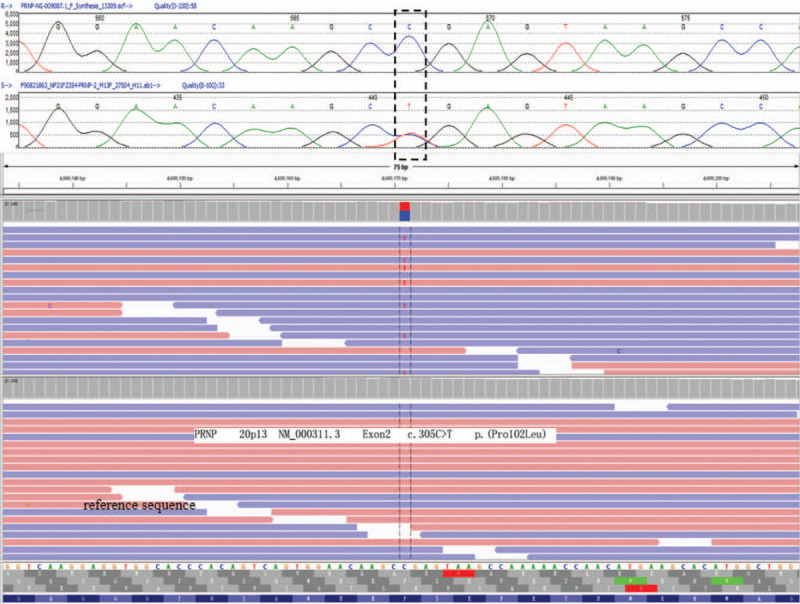
The P102L pathogenic mutation in the prion protein gene. The rectangle shows the precise position of the pathogenic variation on the sequencing result. A missense mutation (C to T) was identified, leading to a proline-to-leucine change at codon 102.

Heterozygous mutations in exon 2 of *PRNP*, positioned on chromosome 20, were detected in our patient. Specifically, a missense mutation (substitution of cytosine with thymine) at gene loci 305 caused a proline-to-leucine substitution (P102L) on the protein polypeptide chain (Fig. 2). Among the GSS-associated mutations, the point mutation at codon 102 is the most common.^[5,6]^ The most common (>80% of cases) and the first discovered mutation in *PRNP* was P102L.^[7]^

Patients with GSS are clinically characterized by the initial adult-onset ataxia and later develop dysarthria, postural abnormalities, dysphagia, and cognitive decline,^[4,6]^ similar to our patient. Patients usually present with ataxia, especially of gait; pyramidal and pseudobulbar signs are easy to find. The MMSE and MRS scores of our patients decreased progressively every year. Remarkable phenotypic heterogeneity has been observed in patients with GSS, and other genetic or environmental factors might be involved in the phenotypic heterogeneity of GSS.^[4]^ Even within 1 family, people with the same gene mutation can show very different phenotypes.

Relatively deep sulcuses in the cerebellar hemisphere and lateral ventricle enlargement in our patient became more apparent, suggesting diffuse cerebral and cerebellar atrophy. At present, most of the neuroimaging studies on GSS have been conducted on small samples (<15 cases). MRI studies have shown mild cerebellar atrophy or diffuse cortical atrophy ^[5]^ in patients with GSS. Three of the 15 MRIs showed a degree of generalized cerebral atrophy, and another MRI had localized cerebellar atrophy.^[8]^ Among patients with significant cognitive or psychiatric features, 3 had generalized atrophy, and 1 had cerebellar atrophy alone.^[9]^ Based on our patient's findings, we hypothesized that cerebral and/or cerebellar atrophy might be a neuroimaging feature of symptomatic GSS. MRIs of patients with GSS showed brain atrophy and demyelinating lesions^[8]^ and restricted diffusion.^[3]^ Of the 7 GSS patients’ MRIs, only 1 showed a high signal in the neocortex, limbic, and striatum on diffusion-weighted images, and 2 had limbic hyperintensities on Fluid-attenuated inversion recovery (FLAIR) and DWI.^[10]^ In 4 of the 15 patients with GSS, the MRI showed multiple white matter lesions.^[8]^ Some of the above patients showed findings resembling small vessel ischemic vascular disease, hyper-intense signal in the caudatum and cortical ribboning, right-sided prevalent anterior cingulate, and insula and bilateral lateral temporal lobe cortical ribboning on DWI and FLAIR images.^[3]^ The imaging findings mentioned above were difficult to distinguish from those of sporadic CJD.

Electroencephalography reveals triphasic periodic complexes ^[3]^ in patients with GSS, although the probability of the wave is <35%.^[11]^ Most electroencephalograms show no specific changes in patients with GSS (such as in our patient), and epileptic waves are rare.^[12]^ In patients with GSS, all diagnostic evaluations have low sensitivity,^[13]^ except high-throughput genome sequencing.

GSS can look not only like other neurodegenerative diseases (i.e., Multiple system atrophy, Alzheimer's disease, and others), but also like CSM. CSM is the most common cause of myelopathy in adults.^[14]^ CSM often develops insidiously and is characterized by clumsiness of the hands, gait instability,^[15]^ lower extremity stiffness, weakness in upper and lower limbs,^[14,16]^ and gait dysfunction including stiff or spastic gait,^[17]^ all of which can also be seen in GSS, hence it is easily misdiagnosed. Other symptoms of CSM include neck stiffness, sphincter disturbances, numbness of the limbs, Lhermitte sign, arm pain.^[14,16]^ These symptoms are useful for a differential diagnosis.

Treatments for GSS may be ineffective unless given in the earliest stages of disease before substantial neuronal damage has occurred.^[18]^ There is no effective treatment of GSS by now, so early diagnosis and subsequent management and clinical care are critical for the patients.^[4]^ A study on a transgenic mouse model for GSS indicated that intravenous immunoglobulin may be a potential effective therapeutic treatment for GSS.^[19]^ GSS usually has a poor prognosis. The disease progression extends over a period of 3.5 to 9.5 years^[20]^ and the mean disease duration is close to 5 years.^[9]^ The early detection of the *PRNP* mutation in patients with GSS might be helpful for prenatal diagnosis and prevention of affected offspring.

Dynamic changes in MRI and quality of life in patients with GSS have rarely been reported. Unfortunately, the family members refused gene detection and the patient refused a brain biopsy.

In summary, cerebral and/or cerebellar atrophy may be a neuroimaging feature of symptomatic GSS. It progresses over time and maybe positively correlated to the severity of symptoms and quality of life. Physicians should consider the possibility of GSS in middle-aged patients with progressive ataxia, cognitive impairment or dysarthria, and brain atrophy on MRI. In addition, it is very important to complete the gene examination.

## Acknowledgments

We thank Editage for English language editing.

## Author contributions

**Conceptualization:** Liming Cao.

**Funding acquisition:** Yanxia Zhou.

**Methodology:** Hongye Feng.

**Project administration:** Jiamei Yi.

**Supervision:** Xuming Huang.

**Writing – original draft:** Liming Cao.

**Writing – review & editing:** Liming Cao.
